# Long-term memory stabilized by noise-induced rehearsal

**DOI:** 10.1186/1471-2202-14-S1-P220

**Published:** 2013-07-08

**Authors:** Yi Wei, Alexei Koulakov

**Affiliations:** 1 Cold Spring Harbor Laboratory, Cold Spring Harbor, NY, 11724, USA

## 

**Figure 1:**
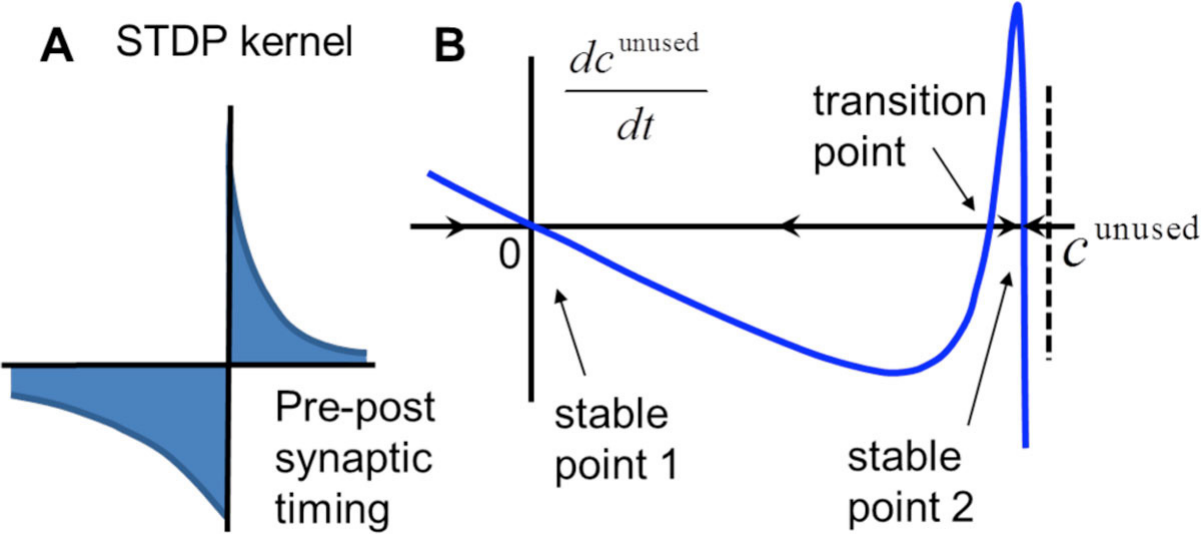
**The stabilization of old (unused) memory states by the combination of unstructured noise and antisymmetric STDP learning rule**. (A) STDP learning rule considered. (B) The rate of change of the contribution of an unused state ($dc^{\text{unused}}/dt$) to the synaptic weight matrix as a function of the contribution itself ($c^{\text{unused}}$). The contribution in this case has two stable points, near zero and at a finite value. The former/latter stable points correspond to the unused memory pattern being absent/present in the network connectivity. At the stable points the rate of change of the pattern's contribution is zero. Small perturbations from the stable point will induce the rate of change that returns the system back to the stable point. The third point where the rate of change is zero is unstable and is therefore called the transition point.

Cortical networks can maintain memories for decades, despite short lifetime of synaptic strength. Can a neural network store long-lasting memories in unreliable synapses? Here we study the effects of random noise on the stability of memory stored in synapses of an attractor neural network. The model includes ongoing spike timing dependent plasticity (STDP). We show that certain class of STDP rules can lead to stabilization of memory patterns stored in the network. The stabilization results from rehearsals induced by noise. We show that unstructured neural noise, after passing through the recurrent network weights, carries the imprint of all of the memory patterns in temporal correlations. Under certain strict conditions, STDP combined with these correlations can lead to reinforcement of all of the existing patterns, even those that are never explicitly visited, i.e. unused. We show that stabilization of unused memories occurs for asymmetric STDP learning rules (Figure 1), while symmetric non-negative rules do not have this property. Thus, we propose that, unstructured neural noise can stabilize the existing structure of synaptic connectivity. Our findings may provide the functional reason for highly irregular spiking displayed by cortical neurons and provide justification for models of system memory consolidation. Out theory makes experimentally testable predictions, such as that synaptic strengths in the cortex should be correlated with the correlations in the pre- and postsynaptic neural activity on the synapse-by-synapse basis. We thus propose that unreliable neural activity is the feature that helps cortical networks maintain stable connections.

